# Midterm results of coracoclavicular stabilization with double augmentation for acute acromioclavicular dislocation

**DOI:** 10.1186/s40064-016-3527-0

**Published:** 2016-10-22

**Authors:** Sungwook Choi, Tong-Joo Lee, Myung-Ku Kim, Ji Eun Park, Hyunseong Kang

**Affiliations:** 1 Department of Orthopaedic Surgery, Jeju National University School of Medicine, Aran 13gil 15, Jeju, 690-767 South Korea; 2 Department of Orthopaedic Surgery, Inha University Hospital, 27, Inhang-ro, Jung-gu, Incheon, South Korea

**Keywords:** Acromioclavicular joint dislocation, Coracoclavicular stabilization, Anatomic reconstruction, Double augmentation

## Abstract

**Introduction:**

Numerous techniques have been introduced for the treatment of acute acromioclavicular (AC) joint dislocation. We aim to report the midterm results of coracoclavicular (CC) stabilization with double augmentation for the acute AC joint dislocation.

**Case description:**

Forty-three patients who underwent surgery for acute AC joint dislocation were followed up for an average of 59.6 months (range 40–97). The study composed of two treatment groups: group S, with 25 patients, in whom two suture anchors were used; and group B, with 18 patients, in whom a suture anchor and a double flip-button device were used, however the techniques in both groups are based on the same principle which is double augmentation. Postoperative evaluations were made retrospectively, clinically, and radiographically.

**Discussion and Evaluation:**

At the last follow-up, the mean Constant score was 91.2 (range 74–100) and the UCLA scale was 31.4 (range 24–35). The overall ratio of the CC distance in the injured shoulder to that in the uninjured shoulder, expressed as a percentage, significantly decreased, to 93.4 ± 22.7 %, immediate postoperatively, and significantly increased, to 113.8 ± 23.4 %, at the final follow-up. Complete reduction of the AC joint was achieved in 34 patients (79.1 %), and 8 patients (18.6 %) exhibited a slight loss of reduction, although their functional outcomes were good.

**Conclusions:**

The results of this study provide evidence that double augmentation is effective in the treatment of acute AC dislocation.

**Level of evidence:**

Therapeutic study, case series, Level IV.

## Background

The incidence of acromioclavicular (AC) joint dislocation is increasing for a variety of reasons. The most common traumatic mechanism is a direct fall on the shoulder with the arm in adduction.

The treatment option selected depends on the severity of the dislocation. Surgical treatment may be considered for acute AC joint dislocations classified as Rockwood grades IV to VI and for acute Rockwood grade III injuries among younger, active patients, particularly high-level athletes and manual laborers (Lemos 1998).

The treatment of Rockwood grade III dislocations is a subject of debate (Dimakopoulos et al. 2006; Murena et al. 2013); current treatments range from functional treatments to complex surgical repairs (Mazzocca et al. 2007). Non-operative treatment often results in excellent clinical outcome and painless shoulder function, although some patients may suffer from chronic instability and pain (Calvo et al. 2006; Taft et al. 1987). According to a study by Deshmukh et al., no difference was observed between the two interventions in terms of strength, pain, and throwing ability (Smith et al. 2011). Thus, we reasoned that Rockwood grade III injuries were excluded from this study.

The goal of treatment for acute AC joint dislocation should be to return the patient to the level of function that he or she enjoyed prior to the injury, with a pain-free, strong and mobile shoulder. However, the ideal treatment for AC joint dislocation is currently controversial. Although numerous techniques have been introduced for the treatment of acute AC joint dislocation (Chernchujit et al. 2006; Choi et al. 2008; Greiner et al. 2009; Mazzocca et al. 2006; Rockwood et al. 1996; Scheibel et al. 2011; Shin et al. 2009; Tienen et al. 2003), the optimal management of this injury remains a subject of debate. Among various surgical techniques, recent studies have reported that the anatomic reconstruction technique is a physiologically viable method that could be used in AC joint reconstruction to produce results comparable to normal joints (Walz et al. 2008; Defoort and Verborgt 2010; Murena et al. 2009; Ladermann et al. 2013).

We have reported previously that minimally invasive coracoclavicular (CC) stabilization with two suture anchors is effective in the treatment of acute AC dislocation (Choi et al. 2008). This prior study demonstrated good results and good patient satisfaction, as well (Choi et al. 2008). Thus, regardless of the implant type, we considered double augmentation an effective technique for AC joint dislocation correction and consistently applied it in our current study. We hypothesized that the midterm results of our study would be as satisfactory as results of previous studies.

## Methods

This is a retrospective study involving a total of 43 patients with grade 4 or 5 acute AC joint dislocations. Forty-three patients who underwent surgery using a suture anchor or a double flip button for acute AC joint dislocation were followed up for an average 59.6 months (range 40–97). These patients were enrolled from among 50 patients with a diagnosis of AC dislocation who underwent surgery between June 2005 and December 2010. A total of 7 of the 50 patients were lost to follow-up and were excluded from this study. After excluding the 5 cases of grade III injuries among the 50 cases, 2 cases were lost to follow-up. The following study was approved by the Institutional Review Board (JNUH IRB File No.: 2013-04-004).

All medical data were reviewed retrospectively. Follow-up data were obtained using questionnaires and by performing physical and radiographic shoulder examinations. Preoperative and intraoperative records were available for all patients.

The study group consisted of 40 males and 3 females whose average age at the time of surgery was 42.6 (range 23–73) years. The patients included both young, active patients [age 20–50, 30 cases (69.8 %)] and relatively older patients [age >50, 13 cases (30.2 %)]. The interval between the time of injury and the date of surgery ranged from 1 to 21 days, with an average of 11.2 days. Eight patients (18.6 %) had Rockwood grade IV injuries, and 35 (81.4 %) patients had grade V injuries.

The injuries were associated with slip-and-fall accidents in 24 (55.8 %) patients, traffic accidents in 10 (23.3 %) patients, and sports injuries in 9 (20.9 %) patients.

Although two types of implants were used in this study, we considered the methods equivalent techniques. The implant groups consisted of group S, treated using 2 corkscrew suture anchors (corkscrew suture anchor with #2 FiberWire and #2 Tigerwire, Arthrex, Naples, Florida, USA), and group B, treated using a corkscrew suture anchor and a double flip-button (DFB) device (TightRope, Arthrex, Naples, Florida, USA). The techniques in both groups are based on the same principle (double augmentation). The only difference between the two techniques was that the latter used a DFB device instead of a suture anchor. All patients were informed of the difference of each implant cost and surgical methods. Implant device was chosen with patient’s consent.

### Clinical evaluations

Every patient was assessed clinically and radiologically after the procedure at a routine clinical follow-up visit. At follow-up, all patients underwent a detailed physical examination for shoulder deformity, AC joint pain on palpation or AC joint pain during cross-arm adduction testing. The evaluation included measurements of pain, activity, range of motion, and strength, and these measurements were recorded using Constant scores (Constant and Murley 1987). The University of California at Los Angeles (UCLA) shoulder rating scale was also used to evaluate patients; this scale is used to assign a score to patients based on their levels of pain, function, active forward flexion, power, and overall satisfaction (Amstutz et al. 1981).

Overall individual satisfaction was rated on a qualitative scale as “very satisfactory”, “satisfactory”, or “unsatisfactory”.

### Radiological evaluation

Initial preoperative radiographs included standard anteroposterior (AP) and axillary views with bilateral stress views to assess the classification of the AC joint separation according to Rockwood et al. (1996). AP stress views and axillary views were obtained for both sides at all follow-ups. The distance between the highest position on the upper surface of the coracoid process and the opposing clavicular undersurface was measured in the AP stress view for both shoulders, yielding the CC distance. The AC distance was measured in an axillary view, and posterior AC displacement was recorded as a negative value. The AC distance was measured from the tip of the clavicular side of the acromion to the anterior corner of the distal end of the clavicle.

In this study, we considered the difference percentages to be more accurate than the actual measurements. Because the radiographic slope and the radiological tester were not standardized, the angle of the beam could be different between radiographs, and thus the actual measurements can differ even for the same patient. Thus, we assumed that comparisons could be made more accurately using the percentage difference between the CC distances of the affected and unaffected sides. Using the Rockwood classification (Rockwood et al. 1996), we have newly defined the extent of reduction as follows: a less than 25 % increase in the CC distance compared to the unaffected shoulder was classified as complete reduction; an increase of 25–90 % in CC distance compared to the unaffected shoulder was classified as a slight reduction loss, and a greater than 90 % increase in CC distance was classified as a complete reduction loss.

### Surgical techniques

The surgical techniques used were described in our previous study (Choi et al. 2008). The patient was placed in a semi-sitting position under general anesthesia. A skin incision was made, the coracoid process was located, and the clavicle was then prepared. After a manual reduction was achieved, the anatomical positions of the conoid and trapezoid ligaments were marked with a K-wire (Rios et al. 2007). Two drill holes were made along the course marked by the K-wire. Two holes were drilled through the clavicle using a 2-mm drill bit, and other holes were drilled through the coracoid for the anatomical replacement of the conoid and trapezoid ligaments. The decision to use an anchor or DFB at the time of repair was based on the surgeon’s preference or the requirements of medical insurance policies. These devices were placed into the base of the coracoid process following the placement of guide pins.

**Fig. 1:**
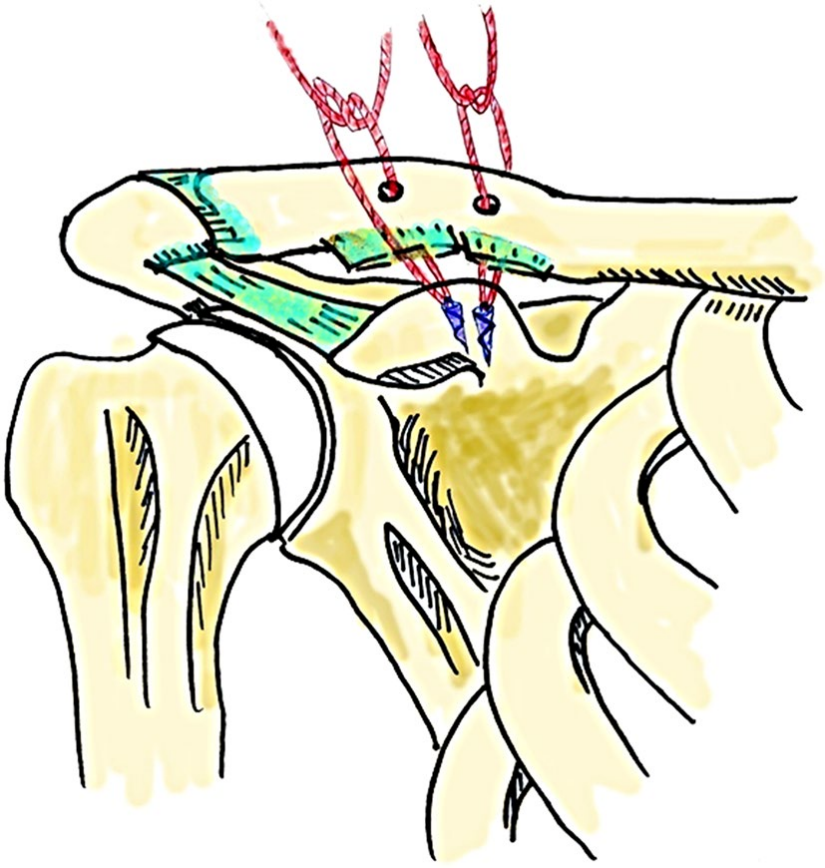
The schema shows the position of the two corkscrew suture anchors in the base of the coracoid process

In group S, two suture anchors were inserted into the attachment sites for the conoid and trapezoid ligaments (Fig. 1).

**Fig. 2:**
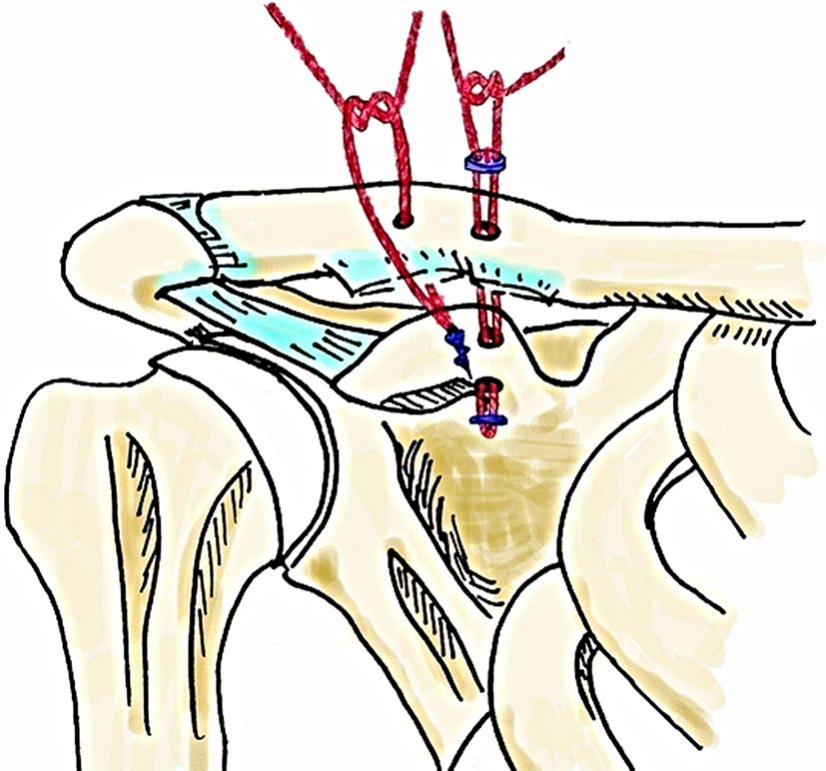
The schema shows the position of the double flip-button device instead of the medial suture anchor

In group B, the trapezoid ligament was replaced with one suture anchor, and the conoid ligament was replaced with one DFB device instead of the medial suture anchor (Fig. 2). After a 3.5-mm hole was drilled at the base of the coracoid process, roll-wire was introduced from the clavicle to the coracoid. With suture thread being relayed from the DFB device, the thread was pulled upward to position the DFB device beneath the coracoid. The suture threads emerging from both sides of the DFB device were then pulled to secure the DFB device to the inferior margin of the base of the coracoid. Firm fixation of the DFB device was performed at the attachment sites described for group S.

### Rehabilitation

Postoperative rehabilitation began immediately, with pendulum exercises. After patients had performed these exercises for 7 days, continuous passive motion (CPM, ORMED gmbh, Freiburg, Germany) exercise was initiated with the goal of attaining a full range of motion in the joint within 8 weeks. After the first postoperative week, assisted active forward flexion exercise using the contralateral arm was permitted and encouraged in the supine position. The arm sling was removed at 8 weeks, and flexion and abduction over 90° were allowed. At 8 weeks, gradual resistance exercises were begun to enhance muscle power. However, heavy lifting was avoided for at least 12 weeks.

### Statistical analysis

All statistical analyses were conducted with the SPSS software package (Version 18.0; SPSS Inc., Chicago, Illinois). Paired t tests were performed to assess the differences in functional scores between the preoperative and postoperative results. Mann–Whitney U tests were performed for comparisons between groups. A p value <0.05 was considered to indicate statistical significance.

## Results

### Clinical results

The average follow-up interval was 59.6 months (range 40–97 months). Except for 2 cases, all patients considered their results to be very satisfactory [n = 26 (60.5 %)] or satisfactory [n = 15 (34.9 %)].

At the last follow-up, the mean Constant score was 91.2 (range 74–100), and the UCLA scale was 31.4 (range 24–35).

One case of suture breakage occurred three months postoperatively, leading to recurrence of the deformity. The suture breakage necessitated revision surgery with open reduction and CA ligament transposition using the Weaver-Dunn technique. A painless range of motion of the injured shoulder was achieved in all patients except one, and no significant functional impairment indicating scapular dyskinesia was noted.

### Radiological results

In AP stress views, the average overall CC distance on the injured side was 19.7 ± 5.2 mm (range 12.0–28.8) preoperatively. The ratio of the measured CC distance to the contralateral equivalent value, expressed as a percentage, was 264.2 ± 51.5 %. The overall CC distance in the injured shoulder fell significantly, to 93.4 ± 22.7 % of that in the uninjured shoulder, immediately postoperatively (p < 0.001).

**Fig. 3:**
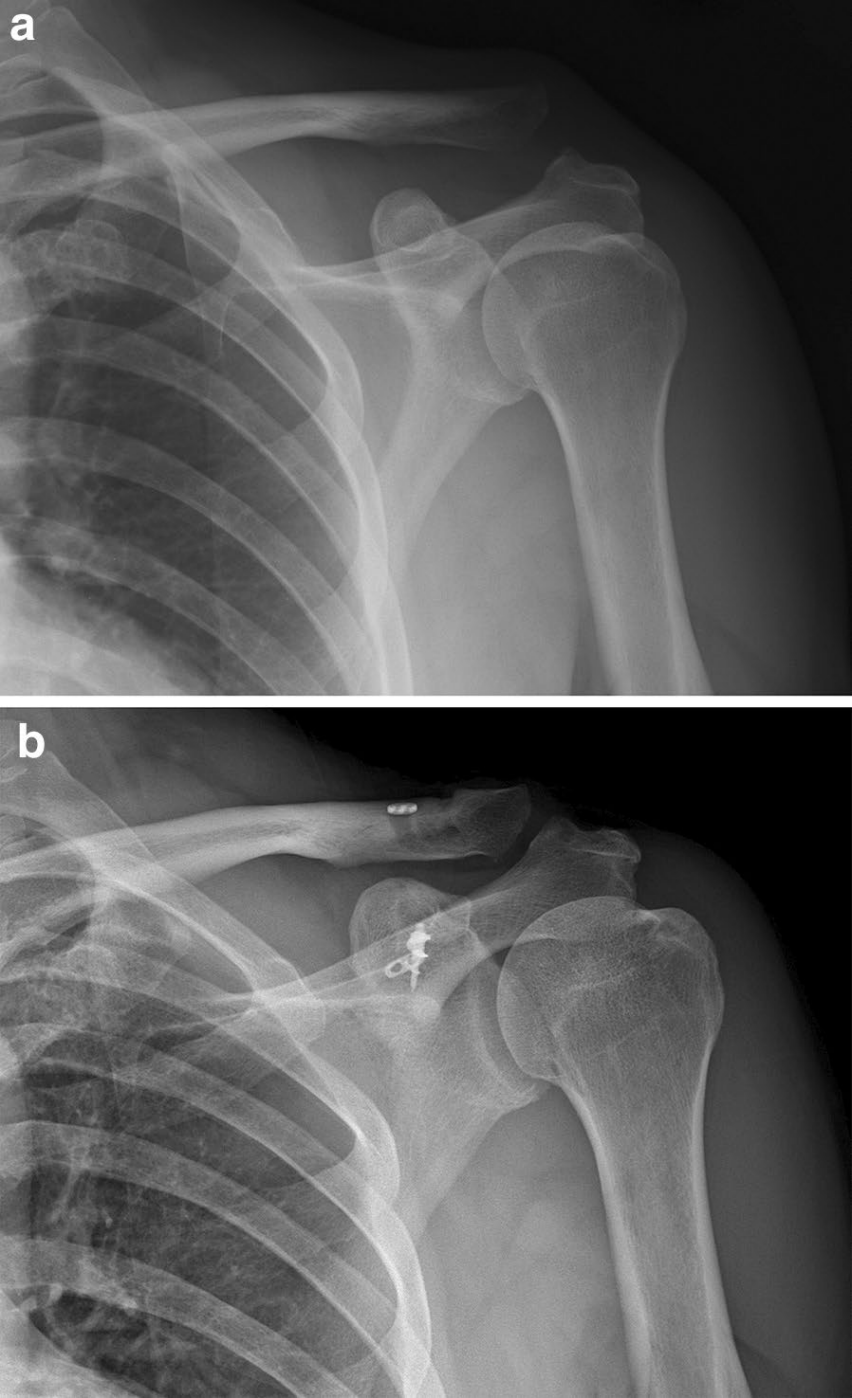
**a** Preoperative anteroposterior radiograph of Rockwood type V injury. **b** 67-months postoperative radiograph shows coracoclavicular interspace remained unchanged with an anchor and a double flip-button

The CC distance was slightly overcorrected immediately postoperatively. However, at the final follow-up, the overall measured CC distance was an average of 8.8 ± 2.4 mm (range 3.4–13.6), and the CC distance in the injured shoulder significantly increased, to 113.8 ± 23.4 % of that in the uninjured shoulder (p < 0.001) (Table 1; Fig. 3).

**Table 1:** Radiologic results

	Total
Preoperative CC distance (mm)	19.7 ± 5.2
Unaffected side CC distance (mm)	7.3 ± 1.8
Preoperative CC distance ratio (%)	264.2 ± 51.5
Postoperative CC distance (mm)	6.8 ± 2.3
Postoperative CC distance ratio (%)	93.4 ± 22.7
F/U CC distance (mm)	8.8 ± 2.4
F/U CC distance ratio (%)	113.8 ± 23.4

$$ {\text{CC}}\;{\text{distance}}\;{\text{ratio}} = \left( {{\text{injured}}\;{\text{shoulder}}\;{\text{CC}}\;{\text{distance}}/{\text{uninjured}}\;{\text{shoulder}}\;{\text{CC}}\;{\text{distance}}} \right)\, \times \,100 $$

*CC* coracoclavicular, *F/U* follow up

In the axillary view, the preoperative and postoperative average AC distances of injured shoulders were −14.3 ± 11.7 mm (range −34.1 to 3.1 mm) and −4.9 ± 9.2 mm (range −19.8 to 5.9 mm), respectively. The average AC distance measured at the final follow-up was −4.3 ± 10.3 mm (range −19.1 to 6.1 mm), which was not statistically significant (p = 0.529).

On radiologic examination (both AP and axillary X-ray views), complete reduction of the AC joint was achieved in 34 patients (79.1 %), and 8 patients (18.6 %) showed slight reduction loss; however, their functional outcomes were good. One patient (2.33 %) had complete loss of reduction due to a car accident.

In 12 patients (27.9 %), postoperative ossification of the CC ligaments was observed, although it did not affect the functional outcome.

### Complications

No neurovascular complications or soft-tissue infections were observed.

## Discussion

Our previous study (Choi et al. 2008) emphasized that minimally invasive anatomic reduction with horizontal and vertical stability is achieved by precisely placing 1 pair of suture anchors in the anatomic position of the CC ligaments, and the current study achieved similar results.

To pass 2 DFB devices through the base of the coracoid process, two 3.5-mm drill holes must be made. However, due to the small anatomy of the coracoid, either 2 suture anchors or one suture anchor and 1 DFB were used. Different anatomical studies of the coracoid have reported the mean coracoid length to be 42.6 ± 0.26 mm (Terra et al. 2013), 45.2 ± 4.1 mm (Rios et al. 2007), and 45.6 ± 4.2 mm (Dolan et al. 2011). However, the insertion site for the DFB or suture anchor is in the anatomical CC ligament attachment region. When the length between the tip of the coracoid and the CC ligament (the osteotomy site for the Latarjet procedure) is subtracted from the total length of the coracoid, the attachment site would be 16.2–24.9 mm long (Rios et al. 2007; Terra et al. 2013; Dolan et al. 2011). These studies were performed in Caucasians or African-Americans. In the Asian population, the mean coracoid length is reported to be 40.5 ± 4.0 mm, and the attachment site is reported to be 10.7–14.7 mm long (Xue et al. 2013). Because the coracoid process is smaller in Asians, two drill holes may overlap or fracture if the distance between them is too close. Thus, we assumed that a dual DFB device insertion technique through two holes was not appropriate for Asians due to this anatomical difference.

Walz et al. (2008) reported the results of fixation using 2 TightRope devices with equal or even higher maximum forces compared with native ligaments. Nuchtern et al. (2013) compared three common procedures (hook plate, TightRope, and bone anchor systems) in an in vitro biomechanical study of AC joint stability. The mean load-to-failure value was 30 % greater in the TightRope group (832.0 ± 401.4 N) compared to the anchor system group (538.0 ± 166.1 N) and was 65 % greater compared to the hook plate group (248.9 ± 72.7 N) (Nuchtern et al. 2013). The TightRope procedure exhibited superior anatomic postoperative displacement (2.04 ± 1.17 mm under a 20-N axial load and 2.83 ± 1.00 mm under a 70-N axial load), whereas the anchor system resulted in moderate translations (5.99 ± 1.89 mm with a 20-N axial load and 6.74 ± 1.98 mm with a 70-N axial load) (Nuchtern et al. 2013).

Although Nuchtern et al. (2013) reported that the load to failure value of tight loop is greater than suture anchor, no statistically significant difference was observed between two groups for reduction in our clinical study [the ratio of CC distance was 98.4 ± 25.1 % in group S and 86.4 ± 17.2 % in group B (p = 0.087)]. We considered that suture anchor and DFB are two different implants. However, in terms of surgical techniques and surgical categorization, they are equivalent.

It is hard to prove the difference of two devices clearly as just our clinical study. If it need that, we thought that the further prospective randomized study about two devices will be required in the future.

If there are patient with small anatomy of coracoid process in acute AC joint dislocation, we recommend that CC stabilization with a suture anchor and a DFB for mechanical stability and anatomical safety.

Overall, patients exhibited slight overcorrection (93.4 ± 22.7 %) immediately postoperatively; this percentage was significantly different at the last follow-up (113.8 ± 23.4 %, p < 0.001). Although the CC distance increased, the results may be presumed to be successful because these lesions may have been clinically asymptomatic.

**Fig. 4:**
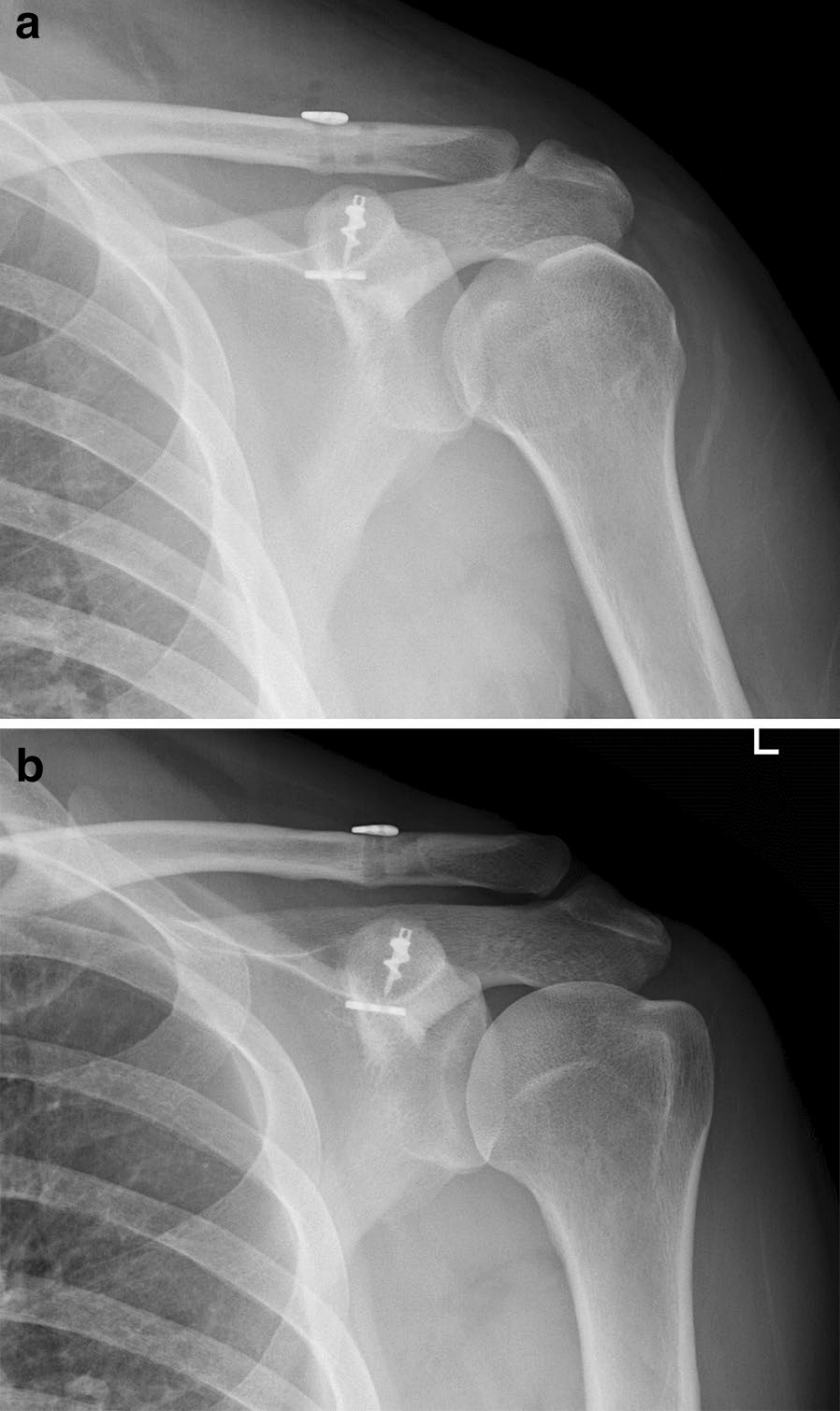
**a** Successful reduction was achieved with an anchor and a double flip-button although immediate postoperative radiograph revealed slight overcorrection of acromioclavicular joint dislocation. **b** Subtle reduction loss was observed at 49-months follow-up

Because the 8 patients with slight reduction loss have good clinical outcomes, these cases may be regarded as Rockwood grade III. All 8 cases exhibited increased CC distances immediately and 1–6 months postoperatively. Further increases in CC distance were not noted at 1-year follow-up. It is assumed that the slight loss of reduction that was observed was generated by the mechanical property of the device strands and that additional reduction losses were no longer observed because the organization of the tissue had become stable (Fig. 4).

Obviously, the goal of surgical treatment is to return the patient to a pre-injury state of joint function. However, a slight reduction loss with a clinically acceptable range of symptoms may also be regarded as a successful surgical outcome. Recently, there has been a trend toward the use of anatomic reconstruction techniques to repair of CC ligaments; these techniques allow for superior primary stability compared with extra-anatomical procedures (Costic et al. 2004; Harris et al. 2000; Jari et al. 2004).

In 12 cases, secondary ossification was observed at the CC interval, and no tenderness was observed at follow-up. We believe that this ossification occurs when bone marrow cells migrate along a torn CC ligament and pass through the tunnels in the bone that were drilled to insert the anchor and the DFB device. These ossifications may not be a complication but instead a structure that may facilitate CC stabilization. Motta et al. (2012) reported that the possible causative factors associated with these ossifications include the transportation of bone fragments by drilling and/or a bone morphogenic protein-mediated process that results in calcium deposition in the soft tissues when the shoulder is at rest.

Double augmentation is used to retain the CC interval rather than to repair the torn ligament, as scar formation will develop around the strands, and ossification will occur to functionally replace the ligament. The approach used to stabilize the joint in the acute phase is to maintain a satisfactory reduction using CC ligament augmentation until the ligaments, particularly the conoid and trapezoid ligaments, heal (Mazzocca et al. 2006; Murena et al. 2009). Ligament reconstructions using the CA ligament (e.g., using the Weaver-Dunn procedure) often appear to be insufficient to stabilize the AC joint, which remains lax in all planes (Deshmukh et al. 2004; Grutter and Petersen 2005). Moreover, such procedures may be criticized because they place the clavicle in a non-anatomic position and because the CA ligament is sacrificed.

Motamedi et al. (2000) found no significant difference in terms of rigidity and resistance between the conoid, the trapezoid, and braided polyethylene (Fiberwire^®^) ligaments. Subluxation of the remnant AC joint does not affect the overall result (Taft et al. 1987). These poor reduction results are evident in radiological images; however, the clinical results for pain and mobility are altered very little or not at all.

A limitation of this study is the small number of cases examined. Although the surgical methods were the same, the difference in the implants may constitute a bias.

We do not consider these slight reduction losses as treatment failures or complications. Although AC joint subluxation was not associated with functional disability of the shoulder joint, precisely locating the sites of anchor insertion should produce excellent results, maintain stability, minimize the risk of subluxation, and thereby increase the chances of achieving complete anatomical reduction. In the present study, all patients showed an excellent functional outcome at the final follow up, and no scapular dyskinesia was reported. Further studies regarding the prevalence of AC joint dislocation-related scapular dyskinesia are required.

Double augmentation using a suture anchor with or without a DFB is a mini-open technique that is easily performed and does not require an additional surgery for device removal. This surgical technique is a useful method of shoulder repair that allows patients to return to their normal activities quickly because it enables early joint motion.
